# Supplementary material for: Deciphering Molecular Factors That Affect Electron Transfer at the Cell Surface of Electroactive Bacteria: The Case of OmcA from Shewanella oneidensis MR-1
Source: Microorganisms. 2022 Dec 28;11(1):79. doi: 10.3390/microorganisms11010079 (PMC9861303; doi:10.3390/microorganisms11010079)
Supplement: Supplementary file 1 [file microorganisms-11-00079-s001.zip › microorganisms-2100154-supplementary.pdf]

Deciphering Molecular Factors That Affect Electron Transfer at the Cell Surface of Electroactive Bacteria: The Case of OmcA from *Shewanella oneidensis* MR-1

Ricardo O. Louro *, Giovanni Rusconi, Bruno M. Fonseca and Catarina M. Paquete *

Supplementary Materials

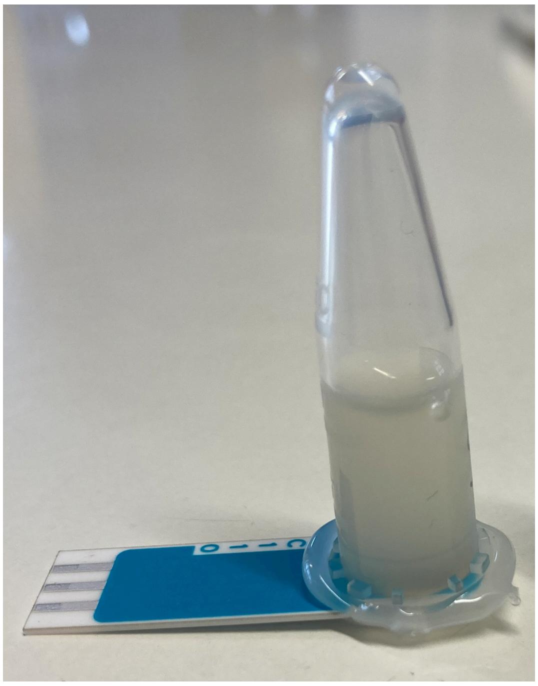

Figure S1. The setup used with the SPEs used to evaluate the electroactivity of the *S. oneidensis* ΔOmcA ΔMtrC cells carrying different OmcA mutants

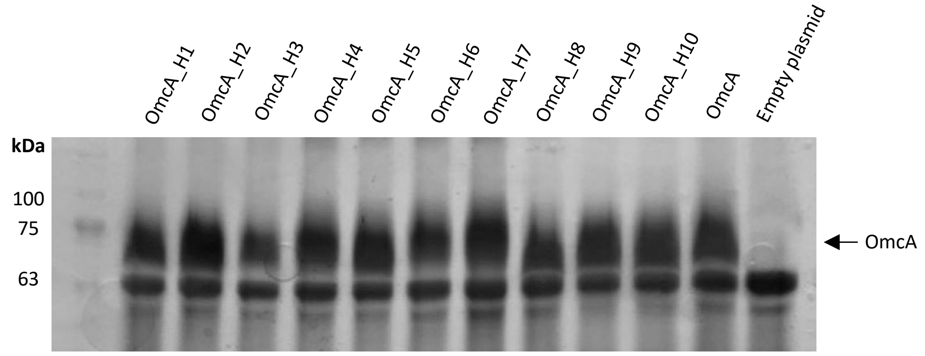

Figure S2. SDS-PAGE of lysed *S. oneidensis* ΔOmcA ΔMtrC cells carrying the different OmcA mutants: SOMR1 ΔOmcA ΔMtrC/pBBR_OmcA H1-H10, native OmcA: SOMR1 ΔOmcA ΔMtrC/pBBR_OmcA and the empty plasmid: SOMR1 ΔOmcA ΔMtrC/pBBR. Toward this, 1 ml of cells grown over-night (to approximately the same OD $_{600\,nm}$) aerobically in LB at 30 ºC at 150 rpm were lysed with Bacterial Cell Lysis Buffer (NZYTech, Portugal), and centrifuged for 20 min at 12 000 x*g*. The same amount of solubilized protein extract was loaded for each strain in the SDS-PAGE. After electrophoresis, the gel was stained for *c*-type cytochromes [47].

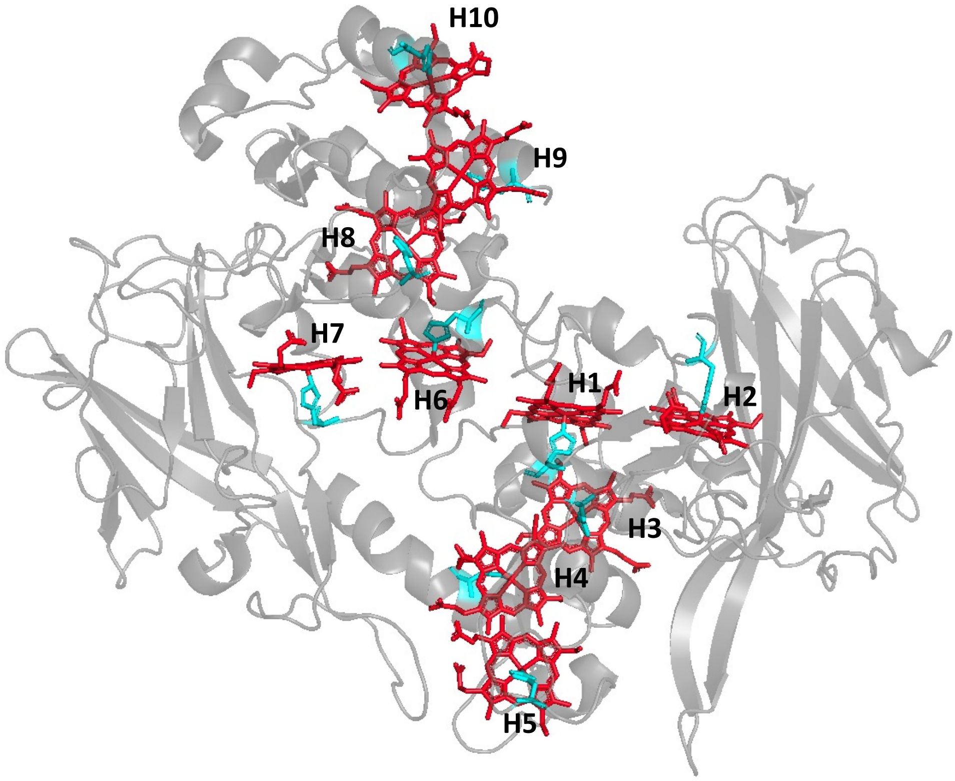

Figure S3. Cartoon representation of the three-dimensional structure of OmcA (PDB 4LMH). The distal histidine residue that was mutated in each individual heme is highlighted in cyan, while hemes are colored in red.

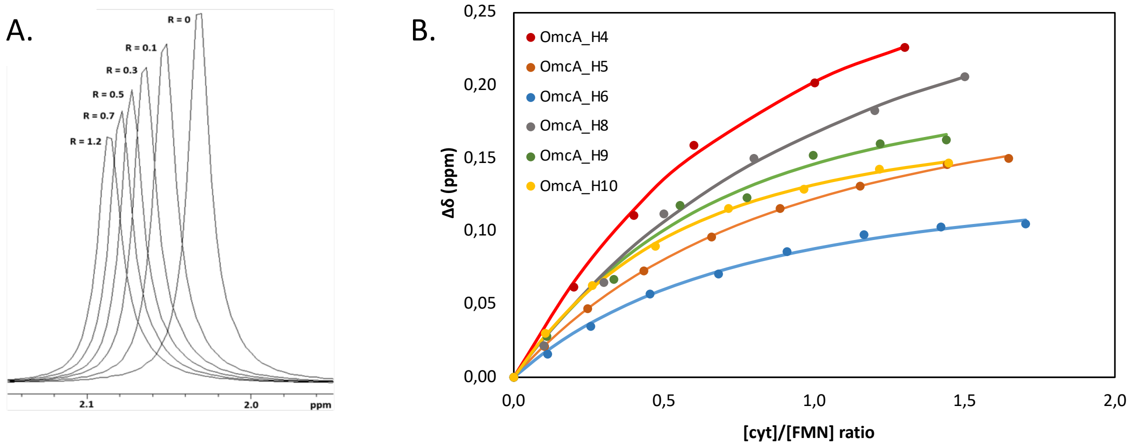

Figure S4. **A.** ^{31}P 1D-NMR spectra of FMN recorded in the presence of increasing amounts of OmcA mutated in heme 10 with R representing the molar ratio of the interaction molecules [OmcA]/[FMN]. **B.** Binding curves of OmcA mutants OmcA_H4, OmcA_H5, OmcA_H6, OmcA_H8, OmcA_H9 and OmcA_H10 with FMN. The chemical shift perturbation of the signal of the phosphorous nucleus of FMN is plotted as a function of the molar ratio of the interacting molecules ([OmcA]/[FMN]). For each mutant the best global fitting for the binding model is represented by a solid line.

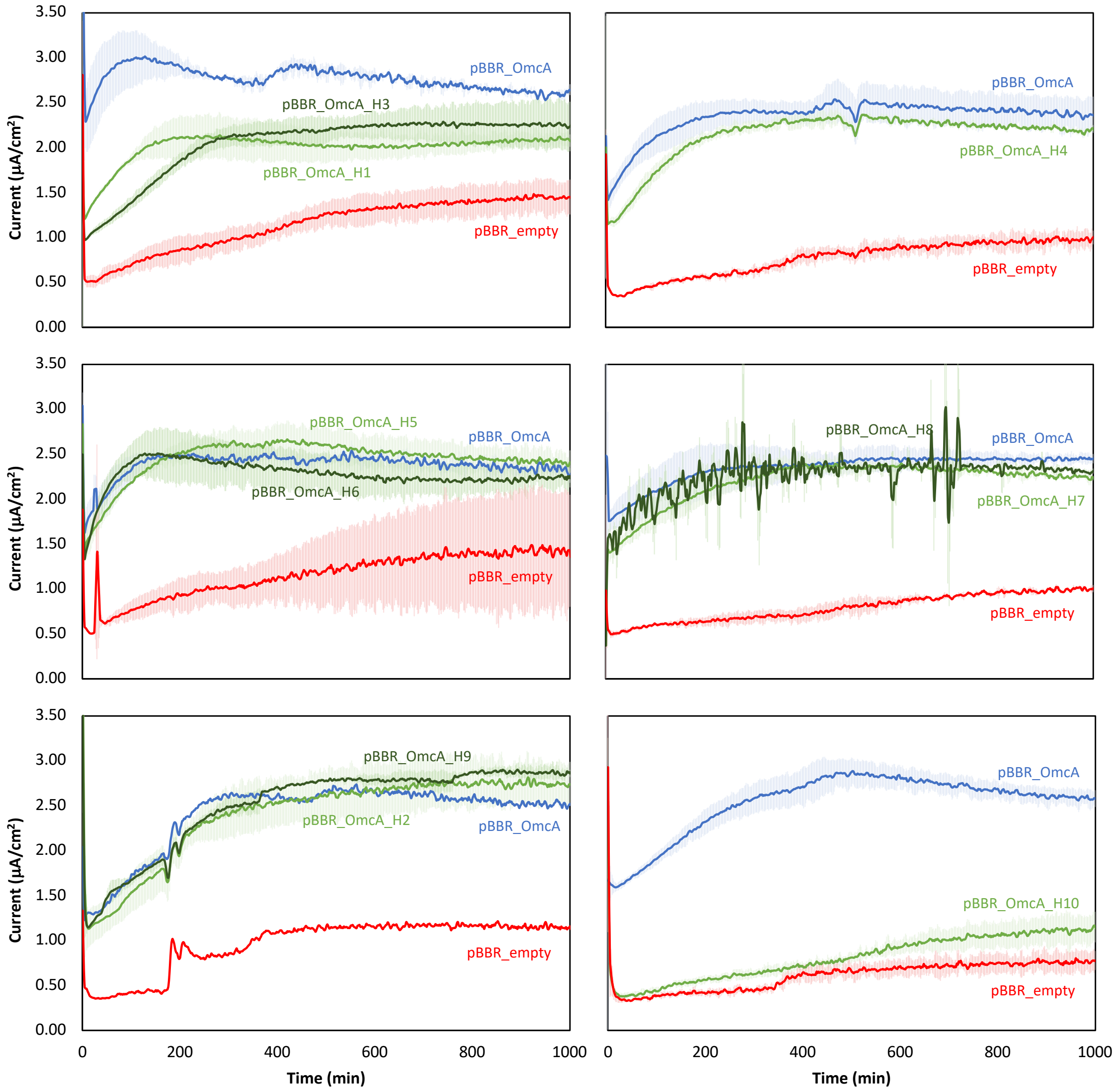

Figure S5. Current density produced by *S. oneidensis* ΔOmcA ΔMtrC cells carrying the different OmcA mutants: SOMR1 ΔOmcA ΔMtrC/pBBR_OmcA H1-H10 (green line), native OmcA: SOMR1 ΔOmcA ΔMtrC/pBBR_OmcA (blue line) and the empty plasmid: SOMR1 ΔOmcA ΔMtrC/pBBR (red line). Error bars shown in the same colors represent the standard deviation of the mean from experiments performed at least in triplicate (except for the native OmcA and empty plasmid of the experiment where OmcA_H2 and OmcA_H9 were tested, with only one experiment represented).

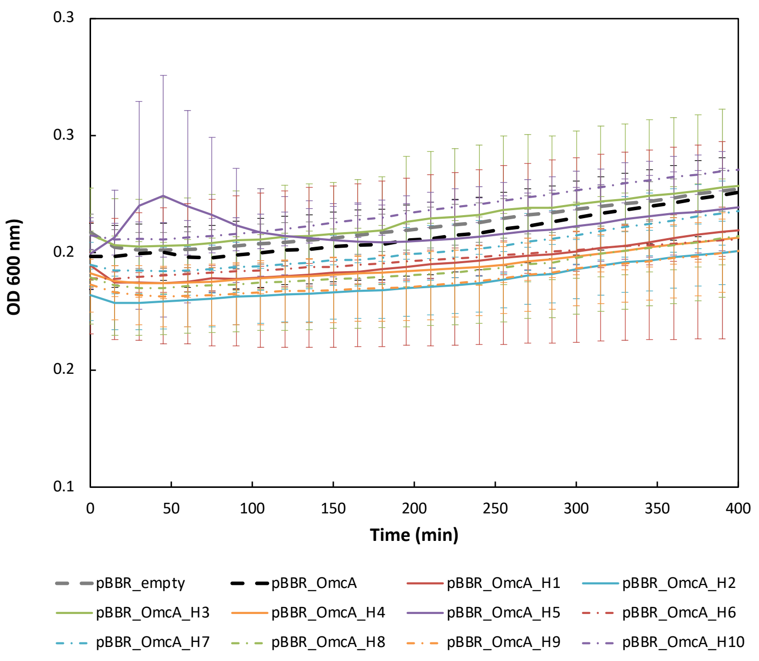

Figure S6. Growth profile of *S. oneidensis* ΔOmcA ΔMtrC carrying different OmcA mutants: SOMR1 ΔOmcA ΔMtrC /pBBR_OmcA H1-H10, native OmcA: SOMR1 ΔOmcA ΔMtrC/pBBR_OmcA and the empty plasmid: SOMR1 ΔOmcA ΔMtrC /pBBR_empty measured at $OD_{600\ nm}$ when the cells are growing under anaerobic conditions in SBM medium at 30 ºC and reducing methyl orange. The error bars represent standard deviations of the measurements.
